# Role of extracellular vesicles in cancer: implications in immunotherapeutic resistance

**DOI:** 10.3389/fimmu.2025.1581635

**Published:** 2025-05-22

**Authors:** Shuai Wang, Zerui Wang, Min Liu, Xiyang Sun

**Affiliations:** ^1^ Emergency Department, Tongren Hospital, Shanghai Jiaotong University School of Medicine, Shanghai, China; ^2^ Wuxi Medical College, Jiangnan University, Wuxi, Jiangsu, China; ^3^ Urology Department, Tongren Hospital, Shanghai Jiao Tong University School of Medicine, Shanghai, China; ^4^ Hongqiao International Institute of Medicine, Tongren Hospital, Shanghai Jiao Tong University School of Medicine, Shanghai, China

**Keywords:** extracellular vesicles, immunotherapeutic resistance, tumor microenvironment, cell communication, cancer progression

## Abstract

Extracellular vesicles (EVs) are lipid membrane-bound vesicles involved in cell-cell communication, particularly in the context of cancer. Immunotherapy, a rapidly evolving field in oncology, is a type of cancer treatment relying on the body’s own immune system to fight mutated cancer cells. Recently, the significance of immunotherapeutic resistance has been increasingly acknowledged owing to the heightened prevalence of cancer and its commonly advanced stage upon diagnosis. However, the complexity and heterogeneity of tumor cells pose challenges to immunotherapy, and the role of EVs in immunotherapeutic resistance remains unclear. Recent studies focused on the role of EVs as heterogeneous groups of nanoparticles in intercellular communication, particularly within the tumor microenvironment (TME). EVs, which include exosomes, shed microvesicles, while apoptotic bodies carry a diverse range of molecular cargo, including proteins, nucleic acids, lipids, and other bioactive molecules. The complexity and versatility of EVs make them a fascinating area of study, with promising implications for the future of immunology and medicine. This brief review highlights the involvement of EVs in immunotherapeutic resistance (e.g., PD-L1 transfer, miRNA-mediated pathways) with a focus on their biogenesis, secretion, and functional roles in cancer, underscoring their potential as diagnostic and therapeutic tools.

## 1 Introduction

Extracellular vesicles (EVs) are membranous particles released by various cell types including cancer cells; they play a pivotal role in intercellular communication (1, 2). EVs mediate cellular signaling by transferring bioactive molecules between cells, thereby modulating cellular behavior and contributing to tumor progression, survival, and metastasis (3, 4). Moreover, cancer cells exploit EVs as vehicles for (5–7) ting drug-resistant proteins or genetic material, thus facilitating the dissemination of resistance mechanisms within the tumor microenvironment and neighboring cells, ultimately leading to therapeutic failure. Furthermore, cancer-derived EVs can modulate the immune response by suppressing the immune cell activity, thereby establishing an immunosuppressive tumor microenvironment that facilitates tumor evasion from immune surveillance (8, 9). Consequently, these effects have garnered increasing attention with respect to immunotherapeutic resistance.

Cancer is a multifaceted disease characterized by dysregulated cellular proliferation and invasive behavior (10). A pivotal hallmark of cancer cells is their capacity to engage in intricate crosstalk with the surrounding microenvironment, including immune cells, stromal cells, and the extracellular matrix (11). This dynamic communication primarily occurs through the secretion of EVs, which exert regulatory effects on recipient cells, thereby modulating and shaping immune response (12, 13). Over the recent decades, extensive research has revealed diverse roles of EVs in cancer biology, particularly in immunotherapeutic resistance (14, 15).

Recent reports offer a comprehensive overview of the complex interplay between EVs and resistance to cancer immunotherapy (16). This highlights the multifaceted nature of this relationship, emphasizing the intricate mechanisms by which EVs influence immune responses and contribute to therapeutic resistance (17).

EVs play sophisticated roles in immune evasion and creation of an immunosuppressive microenvironment (18). Notably, cancer cells utilize EVs to export proteins or antigens, thereby evading immune detection and reducing their susceptibility to immunotherapies that target these specific antigens (19). This insight underscores the cunning tactics employed by cancer cells to subvert the body’s natural defense mechanisms (20). Furthermore, the review points out the significant role of EVs in shaping the tumor microenvironment by delivering immunosuppressive molecules like TGF-β and PD-L1, which inhibits T cell activity and highlights the strategic deployment of EVs to dampen the immune response (21). Additionally, this review delves into the ability of EVs to transfer drug-resistance genes or proteins, leading to the emergence of drug-resistant cancer cell subpopulations. This aspect of EV function is particularly alarming as it suggests a mechanism for the spread of resistance within tumors, complicating the efficacy of immunotherapeutic strategies (22).

In addition, this review discusses the potential of EVs to modulate immune checkpoint molecule expression, which is a critical factor in resistance to immunes (23). This modulation can be further discussed in terms of its implications for the success of immunotherapies that rely on these checkpoints (24). Finally, the review emphasizes the dual role of EVs in instructing immune cells, such as dendritic cells, to either enhance or suppress anti-tumor immune responses. This duality is crucial for understanding how EVs influence the outcomes of adoptive cell transfer therapies to engineer immune cells for the effective targeting of cancer cells (14, 25).

Overall, this article provides a robust foundation for understanding the intricate relationship between EVs and immunotherapeutic (26). A more detailed review could further explore the nuances of this relationship, strategic maneuvers of cancer cells, and challenges posed to the development of effective immunotherapies.

## 2 Subtypes of EVs involved in immunotherapy resistance

**Figure 1:**
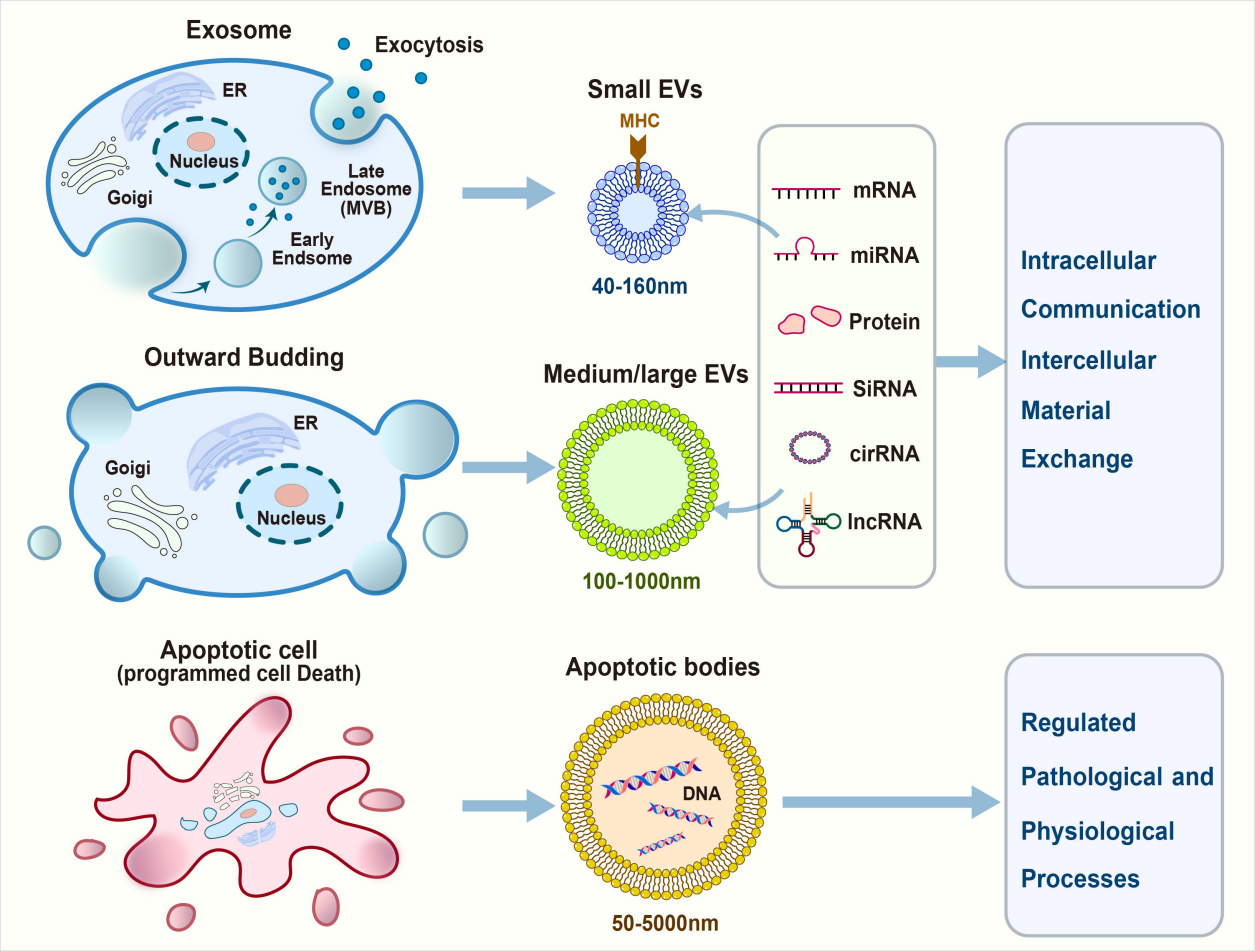
Extracellular vesicles (EVs) communicate with the immune cells in tumor microenvironment.

Based on their biogenesis and biofunctions, EVs are primarily classified into exosomes, shed microvesicles, and apoptotic bodies (**Figure 1**).

### 2.1 Exosomes

Exosomes are small EVs (30–150 nm in diameter) that originate from the endosomal compartment and are generated by the inward budding of the plasma membrane, followed by the formation of multivesicular bodies (MVBs). Subsequently, MVBs merge with the plasma membrane to release exosomes into the extracellular space (27). Exosomes contain several types of specific surface markers, such as tetraspanins (CD9, CD63, and CD81), heat shock proteins (Hsp70 and Hsp90), MVB synthesis proteins (ALG-2-interacting protein X [Alix] and tumor susceptibility gene 101 [Tsg101]), and membrane transporters and fusion proteins (annexins and flotillin) (28). The maturation of MVBs involves the recruitment of specific proteins such as the endosomal sorting complex required for transport, which plays a crucial role in the scission of intraluminal vesicles (ILVs) into the MVB lumen. Exosomes are then released into the extracellular space upon the fusion of MVBs with the plasma membrane (29, 30).

In the intricate tapestry of cancer therapeutics, exosomes have emerged as formidable contributors to immunotherapy resistance in a spectrum of malignancies. Regarding their roles in gynecological cancers, exosomes harvested from cisplatin-resistant ovarian cancer cells encapsulate higher concentrations of cisplatin than their cisplatin-sensitive counterparts, thereby fortifying their resistance to chemotherapy (31). These vesicles are laden with an abundance of drug efflux pumps such as MRP2, ATP7A, and ATP7B, which are instrumental in chemoresistance (32). In breast cancer, adriamycin-resistant cells segregate the drug into exosomes, circumventing the anticipated nuclear accumulation. Moreover, exosomal conveyance of P-glycoprotein (P-gp) induces a chemoresistant phenotype in breast cancer cells (33). Docetaxel-resistant prostate cancer cells release exosomes with increased P-gp levels, outstripping them from their sensitive kin. Exosomes originating from gemcitabine-resistant triple-negative breast cancer cells demonstrate an uncanny ability to impart resistance to more susceptible cells (34). hey also play a role in the development of drug resistance by shuttling proteins, microRNAs, and a plethora of biomolecules capable of modulating therapeutic responses (35).

Engineered exosomes, designed to express the hepatocellular carcinoma antigen α-fetoprotein, have unveiled a potent anti-tumor response by dampening immunosuppressive cytokines and amplifying the presence of IFN-γ-expressing CD8^+^ T cells (36). Exosomes derived from bone marrow mesenchymal stem cells augment chemosensitivity to cisplatin by delivering miR-199a-3p (37), which targets LRRC1 and mitigates drug resistance. Lung cancer cell-derived exosomes orchestrate drug resistance by conveying resistance-associated proteins and RNA. The presence of androgen receptor splice variant 7 within exosomal RNA has been correlated with resistance to hormonal therapy in prostate cancer, underscoring the potential of exosomal biomarkers for predicting treatment outcomes (38–40). Exosomes emanating from cancer-associated fibroblasts (CAFs) play a pivotal role in chemoresistance by fostering colorectal cancer cell stemness and epithelial-mesenchymal transition, which are pivotal in resistance to therapy. The transfer of exosomal miR-92a-3p from CAFs to colorectal cancer cells enhances stemness and epithelial-mesenchymal transition, thereby contributing to chemoresistance to 5-FU/L-OHP (41).

In the discourse on exosomes and their multifaceted roles in cancer, the majority of research underscores their pro-resistance functions; however, few studies have illuminated their contrasting effects. For instance, exosomes from A549 cells, a cisplatin-resistant human lung adenocarcinoma cell line, induce resistance to therapy by upregulating mTOR expression (42). This suggests that targeting the mTOR pathway is a potential strategy to overcome this resistance. However, it’s important to recognize that these effects are not always simultaneous or reversible. In colorectal cancer, exosomes secreted by cancer cells induce resistance to 5-FU and oxaliplatin by activating the Wnt/β-catenin pathway, which promotes the stabilization and nuclear translocation of β-catenin (43). The inhibition of this pathway may be instrumental in reducing drug resistance. Furthermore, triple-negative breast cancer cells release exosomes that induce resistance to docetaxel and gemcitabine in nontumorigenic breast cancer cells (MCF10A). This resistance is mediated by the upregulation of the PI3K/AKT, MAPK, and HIF1A pathways in MCF10A cells (44). In hepatocellular carcinoma, the interaction between the high-mobility group box 1 gene, the RICTOR molecule in the mTOR pathway, and members of the miR-200 family promote glutamine metabolism and tumorigenesis (45). This interaction can reduce the efficacy of anti-PD-L1 immunotherapy in hepatocellular carcinoma. The ORAI1 calcium channel regulates intracellular calcium concentration and affects the secretion of exosomes carrying PD-L1 immune molecules. Silencing the ORAI1 channel in tumor cells inhibits the secretion of PD-L1 exosomes, increases CD8^+^ cells, and impedes tumor progression (46).

These studies highlight the intricate roles of exosomes in modulating immune responses and drug resistance in cancer and shed light on the complexity of exosomal functions and the need for a nuanced understanding of their mechanisms which may pave the way for the development of novel therapeutic interventions.

### 2.2 Shed microvesicles

Shed microvesicles (SMVs), ranging from 100 nm to 1 μm in diameter, directly bud from the plasma membrane. Microvesicles, a subtype of EVs, unlike exosomes, originate without involvement of the endosomal compartment. They are also referred to as the ectosome and originate from outward protrusions of the plasma membrane. Several proteins have been identified as microvesicle-specific, including CD40, ADP-ribosylation factor 6 (ARF6), selectins, phosphatidylserine, and Rho family members.

CAFs secrete EVs rich in annexin A6, which, in gastric cancer cells, stabilize β1 integrin and upregulate the expression of focal adhesion kinase (FAK)-Yes-associated protein (YAP), thereby enhancing cell survival post-cisplatin treatment (47, 48). This suggests a strategic role for EVs in modulating the tumor microenvironment. Furthermore, exosomal transfer of miR-21 from adipocytes to ovarian cancer cells mitigated paclitaxel-induced apoptosis by downregulating apoptotic peptidase activating factor mRNA. This highlights the potential role of EVs in the intercellular communication that influences chemosensitivity. In hepatocellular carcinoma (HCC), sorafenib resistance is induced by the delivery of hepatocyte growth factor (HGF) through EVs, which activates the HGF/c-MET/PI3K/AKT signaling pathway. This pathway activation underscores the significance of EVs in resistance mechanisms and suggests that inhibiting them could be a strategy to mitigate resistance (42, 49).

Macrophage-derived EVs transmit miR-365, which confers gemcitabine resistance to pancreatic adenocarcinoma cells both *in vitro* and *in vivo* (50). This raises the possibility that modulating the cargo of EVs could be a viable approach to overcome drug resistance. CAF-derived exosomal miR-92 significantly promoted T-cell apoptosis and conferred immunotherapy resistance in breast cancer cells. miRNA-92 binds to LATS2, which interacts with YAP1. Chromatin immunoprecipitation confirmed that YAP1 binds to the enhancer region of PD-L1 after nuclear translocation, thereby promoting its transcriptional activity. This revealed a complex regulatory network involving EVs in immune evasion. Hypoxic conditions induce the production and secretion of circEIF3K-containing exosomes from CAFs, which reduces miR-214 expression and upregulates PD-L1 expression, leading to enhanced colorectal cancer cell proliferation, migration, metastasis, and immune escape. This further emphasizes the role of EVs in tumor progression and immune modulation (51, 52).

The following examples highlight the complex interactions between CAFs, EVs, and the tumor microenvironment, and how they contribute to immunosuppression and drug resistance. In EOC, the CAF-derived protein FMO2 facilitates lymphocyte infiltration; higher levels of FMO2 are associated with worse prognosis, suggesting its potential as a biomarker for predicting immunotherapy sensitivity. Hypoxia also induces the secretion of immunosuppressive factors such as TGF-β, VEGF, and PD-L1 from CAFs, which exert an inhibitory effect on T cell-mediated cytotoxicity. This implies the generation of an immunosuppressive microenvironment fostered by CAFs and the potential for targeting these factors to enhance immunotherapeutic outcomes. In colorectal cancer, CD133-containing microvesicles have been identified as promoters of cancer progression by inducing the M2-like polarization of tumor-associated macrophages, a process that could be targeted to combat resistance to immunotherapy. Resistance to sorafenib in invasive HCC cell lines can be attributed to the delivery of HGF via extracellular EVs, which activate the HGF/c-MET/PI3K/AKT signaling pathway. Inhibition of this pathway can potentially mitigate sorafenib resistance (53–55).

Acknowledging the contribution of microvesicles in conferring resistance to immunotherapies is crucial. However, they also present an exceptional opportunity for therapeutic interventions. EVs play a pivotal role in modulating the immune system functions by transporting pro-survival molecules with the potential to induce immune tolerance, thereby facilitating the evasion of immune surveillance by cancer cells (56). Targeting the pro-survival molecules encapsulated within EVs could significantly augment the efficacy of immunotherapeutic approaches. By inhibiting the immunosuppressive effects of microvesicles and these pro-survival molecules, we aim to enhance the effectiveness of immunotherapies and advance personalized and potent cancer treatments.

### 2.3 Apoptotic bodies

Apoptotic bodies, the largest among EVs with sizes ranging from 50 nm to 5 μm, are generated during the twilight hours of apoptosis (57). As the cell undergoes its final stages, its membrane blebs and fragments, releasing bodies laden with cellular remnants. Apoptotic bodies stand out because of their substantial girth compared with their smaller counterparts, exosomes, and microvesicles. Annexin V and histones are apoptotic body-specific proteins (58, 59).

## 3 Biofunction of EVs in cancer

EVs play a pivotal role in the intricate interplay between the immune system and cancer, particularly in their relationship with immunotherapeutic resistance. EVs are small messengers that can influence how our immune cells behave, which is a major concern in cancer growth and spread. They carry various molecules that can either calm the immune response against tumors or create a welcoming environment for cancer cells to thrive. Cancer cells use EVs for their advantage through sending out signals that promote their own growth and spread (15, 60, 61).

The potential of EVs in cancer treatment is an exciting research area. Similar to the creation of a vaccine, EVs can be trained to carry cancer-fighting agents. There is ongoing research to determine whether EVs from dendritic cells can boost the immune response to cancer (62–64). The relationship between EVs and cancer resistance is complex. Cancer cells use EVs to dodge immune attacks, create an environment that suppresses the immune system, and confer resistance to other cancer cells. EVs can also instruct immune cells how to respond to cancer, sometimes by downregulating them (65).

## 4 EVs and immune cells

**Figure 2:**
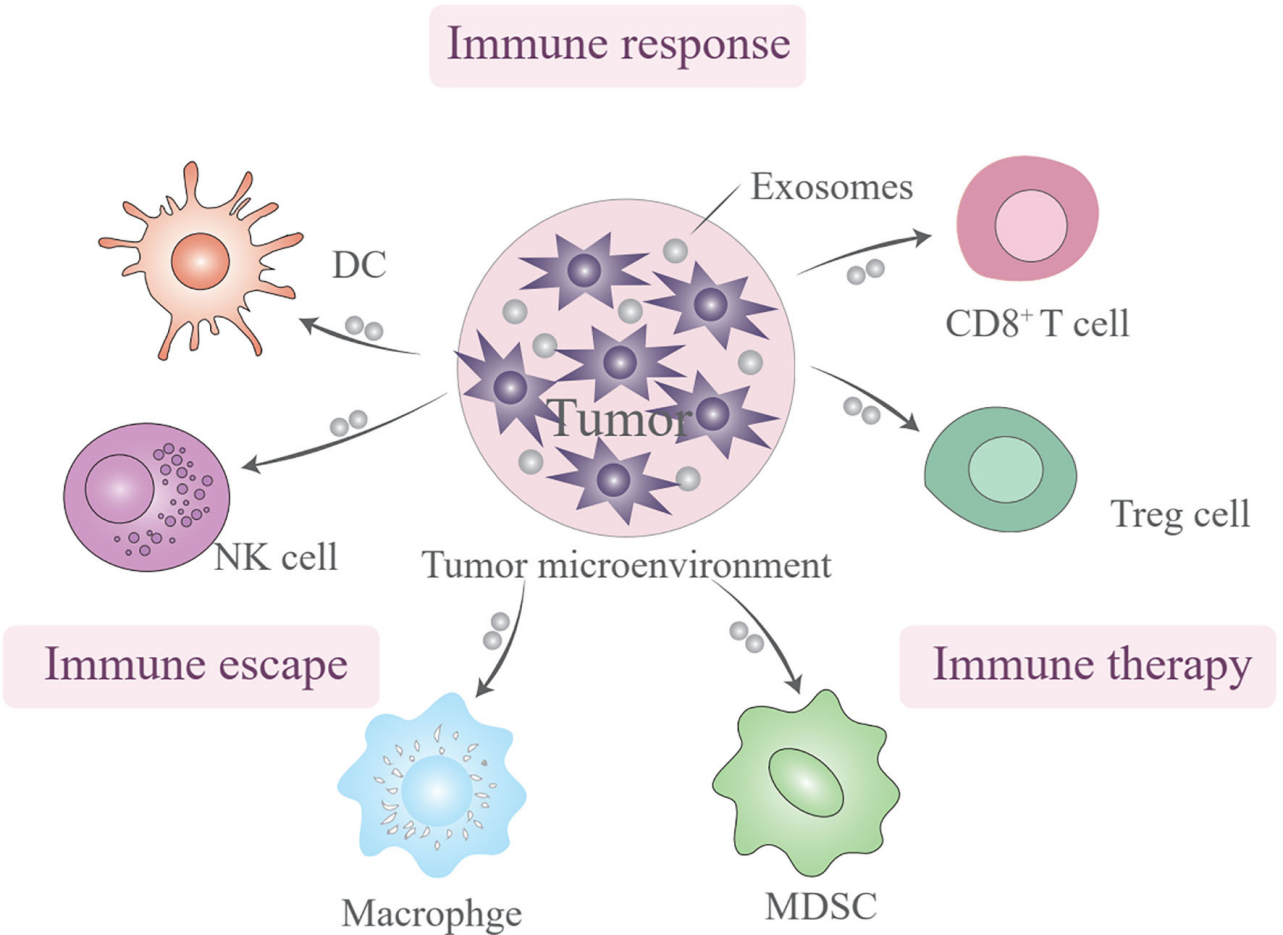
The subtypes of extracellular vesicles (EVs) involved in immunotherapy resistance.

EVs are multifaceted players in the immune system and have the potential to serve as biomarkers and therapeutic agents (66). The influence of EVs on immune cells is a topic of profound interest because these vesicles have a significant impact on both the innate and adaptive arms of the immune system through a myriad mechanisms. EVs are not merely passive bystanders but also active participants in the modulation of immune responses, through activating, suppressing, or even facilitating communication between various immune cells (**Figure 2**). This multifaceted role is particularly evident in the cargo they carry, which reflects their cell of origin and functional state, and encompasses proteins, lipids, miRNAs, and other bioactive molecules that can profoundly influence the activation, differentiation, and effector functions of immune cells (67, 68). EVs should be recognized as pivotal messengers in the immune system, conveying crucial information among immune cells, and thereby fine-tuning immune responses while maintaining delicate homeostasis. The diversity of proteins, nucleic acids, and other bioactive molecules they transport underscores their indispensable role in orchestrating the intricate and tightly regulated processes that underpin immune defenses (69, 70).

Dendritic cells (DCs), which are integral to innate immunity, primarily function as antigen-presenting cells. EVs transfer immune signals between DCs and tumor cells, with potential implications for vaccines targeting tumor immune escape. The presence of phosphatidylserine on the membranes of tumor-derived EVs can bind to CD300a on DCs, reducing IFN-β production and influencing the regulatory T cell (Treg) population. Tumor-associated macrophages and neutrophils (TAMs and TANs), which are prominent scavengers of tumor immunity, communicate with tumor cells through EVs, guide tumor progression, and present promising therapeutic targets. TDEVs facilitate TAM infiltration and participate in establishing an inflammatory immune environment. Finally, EVs act as messengers between tumor cells and natural killer cells, potentially serving as steppingstones in novel therapies (71, 72). EVs play a multifaceted role in the tumor microenvironment (TME), influencing cancer cell behavior and immune response within the tumor microenvironment.

In the realm of immune functions, EVs, especially those derived from antigen-presenting cells, harbor major histocompatibility complex (MHC) molecules that are instrumental in presenting antigens to T cells and modulating adaptive immune responses. This mechanism is of paramount importance for initiating specific immune responses against pathogens or tumors (73, 74). Furthermore, EVs can display immune checkpoint molecules such as programmed death ligand 1 and cytotoxic T lymphocyte antigen 4 on their surface, interacting with receptors on T cells and natural killer cells to inhibit their activity or induce apoptosis, thus playing a critical role in immune evasion in some cancers. It is important to highlight the immunosuppressive properties of certain immune cell-derived EVs. For instance, regulatory T cell-derived EVs (Treg-EVs) carry immunosuppressive molecules like CTLA-4 and TGF-β, which contribute to maintaining immune tolerance and preventing autoimmune reactions, thereby highlighting their role in immune regulation. EVs released by neutrophils, macrophages, and other immune cells contain bioactive molecules such as cytokines, chemokines, and lipid mediators, which can either promote or resolve inflammatory reactions. This implicates them in the pathophysiology of various diseases and positions them as potential therapeutic targets for modulating inflammation (75, 76).

In the context of cancer immunotherapy, EVs have demonstrated their potential to deliver antigens and immunomodulatory molecules to enhance anti-tumor immune responses. A striking example is the release of EVs by chimeric antigen receptor (CAR) T-cells carrying surface CARs, which recognize and induce the death of tumor cells expressing CAR-specific tumor antigens (77).This innovative approach demonstrates the therapeutic potential of EVs in cancer. Moreover, studies have revealed that EVs render target cells more susceptible to inflammatory signals and induce systemic immune responses. They can even render non-responsive cells susceptible to inflammatory agonists, with their inflammatory activity remaining unaffected by soluble receptor antagonists, which is a significant finding for understanding the role of EVs in inflammation (78). A deeper understanding of their biogenesis, cargo, and functional roles is essential to gain insights into the immune system functions and uncover novel therapeutic avenues.

## 5 Immunotherapeutic resistance and EVs

Current immunotherapies mainly focus on the effector arm of the immune system, such as reactivating T cell responses by blocking immune checkpoints, blocking immune checkpoints in cancer immunotherapy, activating adaptive immune responses using tumor vaccines, or directly transferring engineered T cells to tumors (79). For example, Universal immunotherapeutic strategy for hepatocellular carcinoma with exosome vaccines that engage adaptive and innate immune responses (80). Understanding and overcoming this resistance are crucial for improving patient outcomes and the overall success of immunotherapies (81, 82).

The primary reason for overcoming immunotherapeutic resistance is the limited effectiveness of the current immunotherapies. Despite the achieved significant progress in cancer treatment, a substantial number of patients do not respond to immune checkpoint inhibitors or eventually develop resistance, leading to disease progression. First, EVs act as diligent postmen of the TME, shuttling genetic information and various bioactive cargos between different cell types within this microenvironment. This communication network is crucial for maintaining the malignant capacity of tumor cells and plays a significant role in tumor progression and immunotherapeutic dysfunction. For instance, colorectal cancer-derived EVs containing miRNAs can modulate the behavior of recipient cells, thereby influencing the tumor fate. CAFs, a predominant component of stromal cells in the TME, contribute to tumor progression and chemoresistance through their metabolic patterns and secretion of cytokines and chemokines. EVs are indispensable in this reciprocal symbiotic dialogue between tumor cells and CAFs, often converting normal fibroblasts into CAFs through TGF-β or STAT pathways. When we consider the interaction between tumors and endothelial cells, EVs have emerged as initiators of metastasis. They facilitate distant metastasis by affecting the proliferation, migration, and permeability of endothelial cells. For example, in prostate cancer, PGAM1 is transported to human umbilical vein endothelial cells through EVs, thus influencing their metastatic potential (83, 84).

EVs play a role in the resistance to various anticancer therapies. They increase drug efflux, decrease drug toxicity, and enhance DNA repair, contributing to chemoresistance. EVs derived from mesenchymal stem cells contribute to the development of therapeutic resistance to chemoresistance, targeted therapy, and immunotherapy. CAR-T cell activity is suppressed by EVs from cancer-associated fibroblasts (CAFs) that deliver immunosuppressive cytokines (e.g., TGF-β, IL-10), fostering a TME enriched with regulatory T cells (Tregs) and myeloid-derived suppressor cells (MDSCs) (85).EVs act as double-edged swords in these therapies. Tumor-derived EVs (TDEVs) carry functional PD-L1, which binds PD-1 on T cells, mimicking immune checkpoint interactions and blunting ICI efficacy (86). EVs also scavenge tumor-associated antigens (TAAs), reducing antigen availability for dendritic cell (DC) priming and adaptive immune activation. Additionally, The role of the TME in resistance is further complicated by its heterogeneity, with different regions within the same tumor potentially having distinct immune landscapes (87, 88). This heterogeneity can lead to differential responses to therapy and outgrowth of resistant clones.

EVs drive resistance through diverse mechanisms that compromise immune cell function and enhance tumor survival: (1) Immune suppression via cargo transfer, TDEVs deliver immuno suppressive molecules (e.g., TGF-β, adenosine) to expand Tregs and MDSCs, creating an inhibitory TME. Exosomal miR-21 from adipocytes downregulates apoptotic pathways in ovarian cancer cells, conferring resistance to paclitaxel (89). (2) Antigen masking and immune evasion EVs shed TAAs (e.g., HER2, MUC1), reducing antigen visibility and limiting T cell recognition. CAF-derived EVs transfer miR-92a-3p to colorectal cancer cells, promoting stemness and resistance to 5-FU/oxaliplatin by activating Wnt/β-catenin signaling. Exosomal PD-L1 suppresses CD8^+^ T cell activity, while ORAI1 calcium channel inhibition reduces PD-L1 exosome secretion, restoring T cell infiltration (90, 91). (3) Activation of pro-survival pathways: Hepatocyte growth factor (HGF) in EVs activates the HGF/c-MET/PI3K/AKT axis in hepatocellular carcinoma (HCC), driving resistance to sorafenib. (4) Drug Efflux and Metabolic Reprogramming: EVs export chemotherapeutics (e.g., cisplatin) via drug efflux pumps (e.g., MRP2, P-gp). Exosomal circEIF3K from hypoxic CAFs reduces miR-214 levels, upregulating PD-L1 and promoting immune escape in colorectal cancer (92).

Despite their role in resistance, EVs hold promise for improving immunotherapy outcomes through strategic engineering: (1) EV-Based Immune Activation: Dendritic Cell-Derived EVs Loaded with TAAs, these EVs act as vaccines to prime cytotoxic T cells and enhance antigen presentation (93). CAR-T Cell-Derived EVs display CARs on their surface, enabling bystander killing of antigen-negative tumor cells and overcoming tumor heterogeneity (94, 95). (2) Reversing Resistance Mechanisms: silencing exosomal PD-L1 or miR-92a-3p restores T cell activity and checkpoint inhibitor sensitivity (96–98). Engineered EVs carrying CRISPR-Cas9 can knockout resistance genes (e.g., β-catenin) in recipient cells (99). (3) Targeted Delivery Systems: EVs loaded with immunostimulatory molecules (e.g., IFN-γ, IL-12) reprogram the TME to support anti-tumor immunity. Hybrid EVs fused with liposomes enhance drug delivery to tumors while minimizing off-target effects (100).

In summary, the interplay between EVs and immunotherapeutic resistance underscores their dual role as both adversaries and allies in cancer treatment. While EVs facilitate immune evasion and therapy resistance through immunosuppressive cargo and pathway activation, their engineering potential offers innovative strategies to enhance ICIs, CAR-T therapies, and personalized medicine. Future research should focus on deciphering EV heterogeneity and optimizing delivery platforms to fully exploit their therapeutic capabilities (101).

## 6 Conclusion

EVs are multifaceted players in the cancer landscape that influence tumor biology, immune responses, and therapeutic outcomes. The realm of cancer immunotherapy has been invigorated by the advent of EVs, which have emerged as potent vehicles for the delivery of antigens and immunomodulatory molecules, thereby amplifying anti-tumor immune responses. This development is particularly noteworthy, given the remarkable capacity of EVs released by CAR-T cells to carry surface CARs, enabling them to specifically target and induce the death of tumor cells bearing CAR-matching tumor antigens. This innovative strategy not only underscores the therapeutic potential of EVs in oncology but also represents a significant stride in the field of targeted cancer therapies.

Furthermore, the sensitization of target cells to inflammatory signals and eliciting systemic immune responses by EVs are crucial. The ability of EVs to convert nonresponsive cells into inflammatory agonist-susceptible cells, with their inflammatory activity remaining impervious to soluble receptor antagonists, is a groundbreaking finding. This aspect of EV biology is particularly intriguing as it sheds light on its role in inflammation and suggests that it may play a key role in modulating immune responses in a variety of pathological contexts.

Further study should confront the following three understudied dimensions to advance EV-based therapeutics: a. Spatiotemporal Heterogeneity of EV Cargo. Current studies predominantly focus on bulk EV analysis, neglecting subpopulation-specific functions. For example, apoptotic bodies versus exosomes exhibit divergent roles in mediating radiation resistance versus chemotherapy tolerance. Single-vesicle profiling technologies could unravel this complexity. b. EV-Driven Metabolic Reprogramming: Emerging evidence suggests that CAF-EVs transfer lactate dehydrogenase A (LDHA) and glutamine synthetase to tumor cells, fostering an acidic, nutrient-depleted microenvironment that impairs T-cell glycolysis and cytotoxicity. c. Host-Microbiota-EV Axis: Gut microbiota-derived EVs modulate systemic immunity by regulating PD-L1 expression on dendritic cells. However, their impact on ICB resistance remains unexplored—a critical omission given the clinical correlation between dysbiosis and immunotherapy failure.

As we delve deeper into the understanding of EV biogenesis, cargo, and functional roles, it becomes increasingly clear that these vesicles are not just passive participants but also active mediators in the complex interplay of the immune system. This deeper understanding is vital for deciphering the intricacies of immune function and paving the way for innovative therapeutic strategies. Harnessing EVs for cancer treatment holds immense promise, and continued research on their mechanisms of action will undoubtedly yield valuable insights and potential clinical applications.
